# Current insights of community mental healthcare for people with severe mental illness: A scoping review

**DOI:** 10.3389/fpsyt.2023.1156235

**Published:** 2023-04-18

**Authors:** Caroline van Genk, Diana Roeg, Maaike van Vugt, Jaap van Weeghel, Tine Van Regenmortel

**Affiliations:** ^1^School of Social and Behavioral Sciences, Tranzo Scientific Center for Care and Wellbeing, Tilburg University, Tilburg, Netherlands; ^2^Kwintes Housing and Rehabilitation Services, Zeist, Netherlands; ^3^Trimbos Institute, Dutch Institute of Mental Health and Addiction, Utrecht, Netherlands; ^4^HVO-Querido, Amsterdam, Netherlands; ^5^Faculty of Social Sciences – HIVA, University of Leuven, Leuven, Belgium

**Keywords:** severe mental illness, community care, recovery, social inclusion, scoping review, current insights, human rights, independent living

## Abstract

**Background:**

For the last four decades, there has been a shift in mental healthcare toward more rehabilitation and following a more humanistic and comprehensive vision on recovery for persons with severe mental illness (SMI). Consequently, many community-based mental healthcare programs and services have been developed internationally. Currently, community mental healthcare is still under development, with a focus on further inclusion of persons with enduring mental health problems. In this review, we aim to provide a comprehensive overview of existing and upcoming community mental healthcare approaches to discover the current vision on the ingredients of community mental healthcare.

**Methods:**

We conducted a scoping review by systematically searching four databases, supplemented with the results of Research Rabbit, a hand-search in reference lists and 10 volumes of two leading journals. We included studies on adults with SMI focusing on stimulating independent living, integrated care, recovery, and social inclusion published in English between January 2011 and December 2022 in peer-reviewed journals.

**Results:**

The search resulted in 56 papers that met the inclusion criteria. Thematic analysis revealed ingredients in 12 areas: multidisciplinary teams; collaboration within and outside the organization; attention to several aspects of health; supporting full citizenship; attention to the recovery of daily life; collaboration with the social network; tailored support; well-trained staff; using digital technologies; housing and living environment; sustainable policies and funding; and reciprocity in relationships.

**Conclusion:**

We found 12 areas of ingredients, including some innovative topics about reciprocity and sustainable policies and funding. There is much attention to individual ingredients for good community-based mental healthcare, but very little is known about their integration and implementation in contemporary, fragmented mental healthcare services. For future studies, we recommend more empirical research on community mental healthcare, as well as further investigation(s) from the social service perspective, and solid research on general terminology about SMI and outpatient support.

## 1. Introduction

For the last three decades, there has been a shift in mental healthcare from a biomedical model to a more biopsychosocial model with a focus on rehabilitation, strengths, all areas of recovery, citizenship, empowerment, autonomy, and shared decision-making as leading principles (1–5). Still, the “social aspect” of the biopsychosocial model has long remained neglected (6). In 2007, human rights for people with disabilities were covered in the convention (7), and several community-based mental healthcare programs and services have been developed in Europe for these groups, enhanced by peer-to-peer initiatives and recovery colleges (8). Over the past few years, concepts such as social inclusion, citizenship, and participation have become the heart of the deinstitutionalization movement. Additionally, more and more people with mental healthcare issues receive outreach support. An indication of the development of intensive outpatient care for people with severe mental illness (SMI) is the development of (flexible) assertive community treatment ((F)ACT) teams. For example, in Netherlands in 2020, there were an estimated 400 FACT teams (9) and about 30% of people with SMI in England receive support from a specialist mental health floating outreach service (10).

In general, the definition of SMI consists of three criteria: a psychiatric diagnosis according to Diagnostic and Statistical Manual of Mental Disorders, illness duration of more than 2 years, and disability in functioning (11). A subgroup of people with SMI needs intensive care and support in daily living and receives residential care, supported housing in a 24/7 facility, or floating outreach (12). Most people with SMI who live in residential supported housing facilities have a strong preference to live independently in the community with flexible support with a view to a meaningful and fulfilling life (13). Nowadays, there are several community-based support services for these people who want to live independently, such as Housing First (HF). HF is an evidence-based housing intervention in the social domain that combats homelessness (14). It combines rapid access to permanent, nonabstinence-contingent ordinary housing and recovery-oriented mental health support teams (15). Individuals with SMI are at a higher risk of homelessness, and a high proportion of individuals experiencing homelessness are also living with mental illness (16). Therefore, measures should be available to prevent those who do not make use of, or leave, supported housing from becoming homeless.

Different services for mental health conditions have traditionally been separate from other services such as physical healthcare and social services. However, there is increasing emphasis internationally on developing a whole-system approach to improve the integration of these services to maximize an individual’s quality of life and social inclusion by encouraging their skills, promoting independence and autonomy to give them hope for the future. That leads to successful community living through appropriate support, with particular focus on patient-centered development and delivery (17–19). Furthermore, following the rehabilitation and recovery movement, care should involve all areas of living (20), and community-based mental healthcare thus should be a more integrated package of services. Many studies have appeared on the development and impact of multidisciplinary teams in mental healthcare (21, 22). A lot less research is available on supported housing services, including accommodation-based and floating outreach services, leading to a lack of evidence on what works in this area (23, 24).

In this literature review, we focus on all services for persons with SMI which are living independently in the community. These services aim to support these people in their daily life. This includes services initiated by treatment organizations, such as ambulatory interdisciplinary teams, as well as by welfare and supported housing organizations. Following McPherson et al. (25), who developed the simple taxonomy for supported accommodation (STAX-SA) to capture the defining features of different supported accommodation models, in this study we focus on supported housing services meant for persons moving forward from a hospital admission or a full-time staffed housing accommodation in a congregate setting with high support, toward more individual accommodation with no staff on-site. These services can be low or might need to be medium or intensive to support independent living for all (25).

Currently, there is a lack of research about what is needed to successfully provide this type of intensive support for people with SMI, and especially about how this support can be organized as an integrated community-based mental healthcare approach, including housing, rehabilitation, citizenship, all areas of recovery, empowerment, autonomy, and decision-making power. We aim to provide a comprehensive overview of existing and upcoming community mental healthcare approaches to discover the current vision and empirical findings on the ingredients of community mental healthcare. To do so, we will look in this review for both empirical evidence, as well as leading concepts in this research topic. The findings of this study contribute to the further development of community-based mental healthcare for persons with SMI and high-volume healthcare needs. This paper will address the following question: What are the current insights (both leading concepts and empirical findings) regarding a community mental healthcare system to support all persons with SMI in their independent living and recovery, and stimulate further social inclusion?

This review follows the PRISMA guidelines for scoping reviews (26). The completed PRISMA checklist is available on request from the authors.

## 2. Methods

### 2.1. Study design

We performed a scoping review, following the steps of the framework of Arksey and O’Malley (27): (a) identify the research question; (b) identify relevant studies; (c) select the studies; (d) chart the data; and (e) collate, summarize and report the results. A scoping review contributes to mapping rapidly the key concepts underpinning a research area and the main sources and types of evidence available (28).

### 2.2. Eligibility criteria

#### 2.2.1. Inclusion criteria

We included papers published in English from January 2011 to December 2022 in peer-reviewed journals, aimed at 18 years and older adults with severe mental illness, focusing on stimulating independent living, integrated care, recovery, and social inclusion. For reasons of comparability, and fit in Western healthcare systems, studies were included if they were conducted in Western countries only (i.e., United States of America, Canada, countries in Western Europe, Australia, New Zealand, and Japan). Finally, all study designs were included, and we also included papers about interventions related to collaboration.

#### 2.2.2. Exclusion criteria

Studies were excluded if (a) they primarily focused on treatment without support or care, (b) social inclusion or recovery was not the aim, (c) they focused on interventions that concentrated on one area of life and did not provide an integrated offering, or (d) if they focused on psychometric or physical diseases.

### 2.3. Search strategy

**Figure 1:**
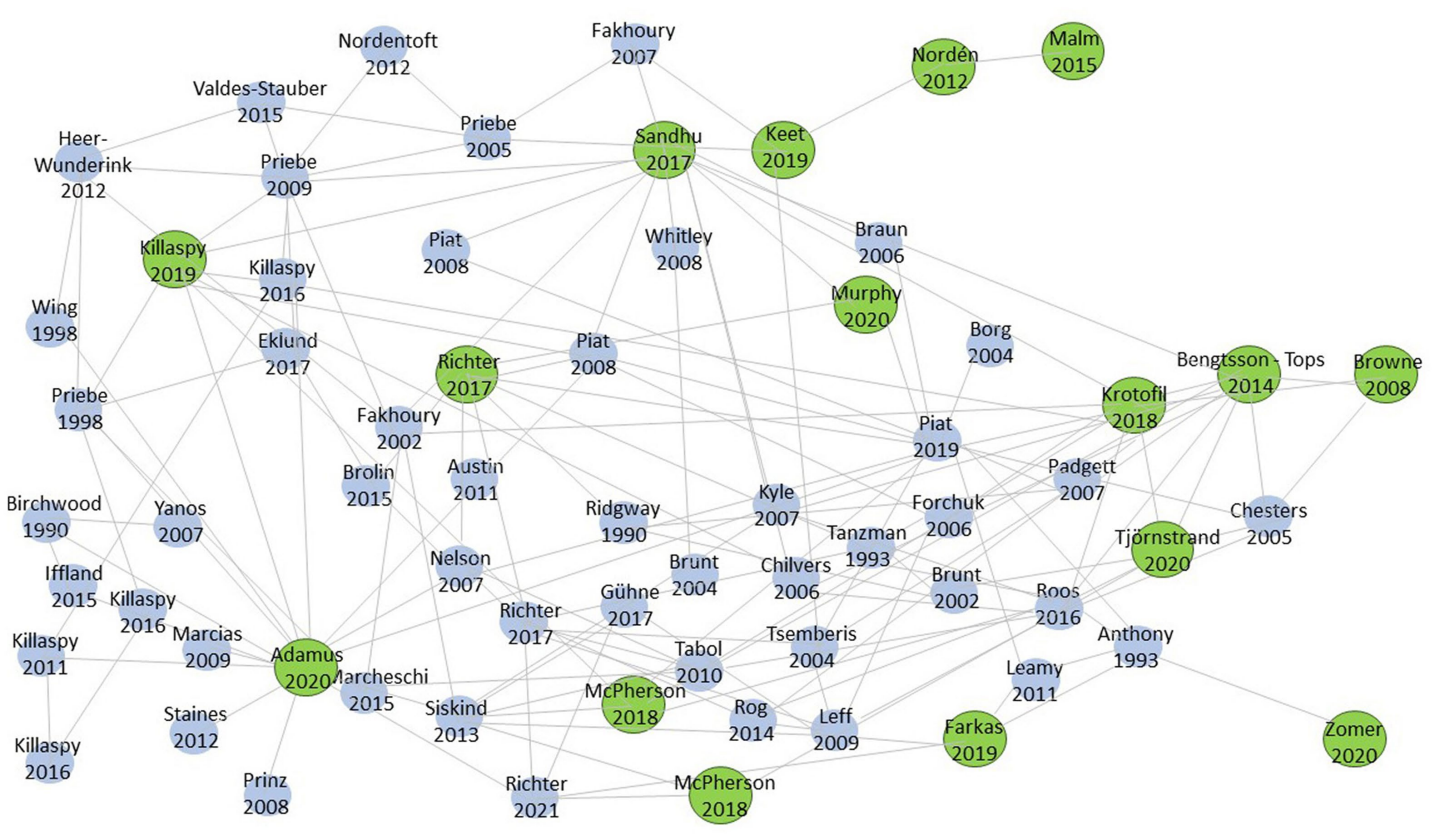
Research Rabbit.

To find the right search terms for our search, we used the program Research Rabbit. This program helps to explore the literature of a research topic and links authors and papers on the same topic to each other. Before conducting the search, the research team determined the eight most relevant papers on this topic and added them to the program. With the function “similar work,” we added another eight relevant papers. Figure 1 shows these 16 relevant papers with the biggest bullets and shows that some papers have more in common with each other than others. The most common keywords from the 16 papers were the basis for our search terms.

We formulated and combined search terms concerning: (a) population (Serious Mental Illness* OR Severe Mental Illness* OR SMI OR Mental Illness OR Psychiatric Disabilities); (b) the setting (Community Mental Health* OR Supported Housing OR Supportive Housing OR Supported Accommodation OR Community-based mental healthcare OR Independent Living OR Independent Housing); (c) outcomes (Recovery OR Psychiatric Rehabilitation OR Rehabilitation OR Participation OR Social Inclusion OR Empowerment); and (d) contemporary paradigm (Deinstitutionali* OR De-Institutionali* OR Community Living OR Integrated Care). To reduce the number of irrelevant studies, exclusion terms based on the eligibility criteria were added to the search strategy (e.g., somatic disease, dementia, and COVID-19).

We systematically searched the following electronic databases: PubMed, PsycInfo, Medline, and Cinahl (September 2021, updated in December 2021 and December 2022). These databases were chosen to cover medical (PubMed and Medline), psychological (PsycInfo), and nursing (Cinahl) literature. After the database search, we reviewed the reference lists from papers included by title and abstract to find missing important papers, and additionally, the volumes of the *Journal of Integrated Care* and the *Community Mental Health Journal* published in the same period (2011–2021) were reviewed. Finally, we added several papers manually in consensus with our research group that were found lacking in the results, but which did meet the inclusion criteria.

### 2.4. Study selection process

After the removal of duplicate papers by the first author, the papers were screened in three rounds. In the title, abstract, full-text screenings phase, and thematic analysis, the first author screened all the hits and the second and third authors screened a random sample of 5% to ensure, and reach consensus on, fidelity to the inclusion criteria.

### 2.5. Data analysis

A qualitative synthesis of included studies was performed using the method of thematic analysis. All papers were screened on elements of relevance (or ingredients needed) for current community mental healthcare with the aim to support persons with SMI in their independent living, recovery, and to stimulate further social inclusion. All papers were coded, and codes were synthesized into areas of ingredients.

## 3. Results

First, we present the descriptives in a PRISMA flow diagram, and a summary of the characteristics and quality of the studies included. Second, we present the results of our qualitative synthesis using thematic analysis.

### 3.1. Flowchart and summary of studies found

**Figure 2:**
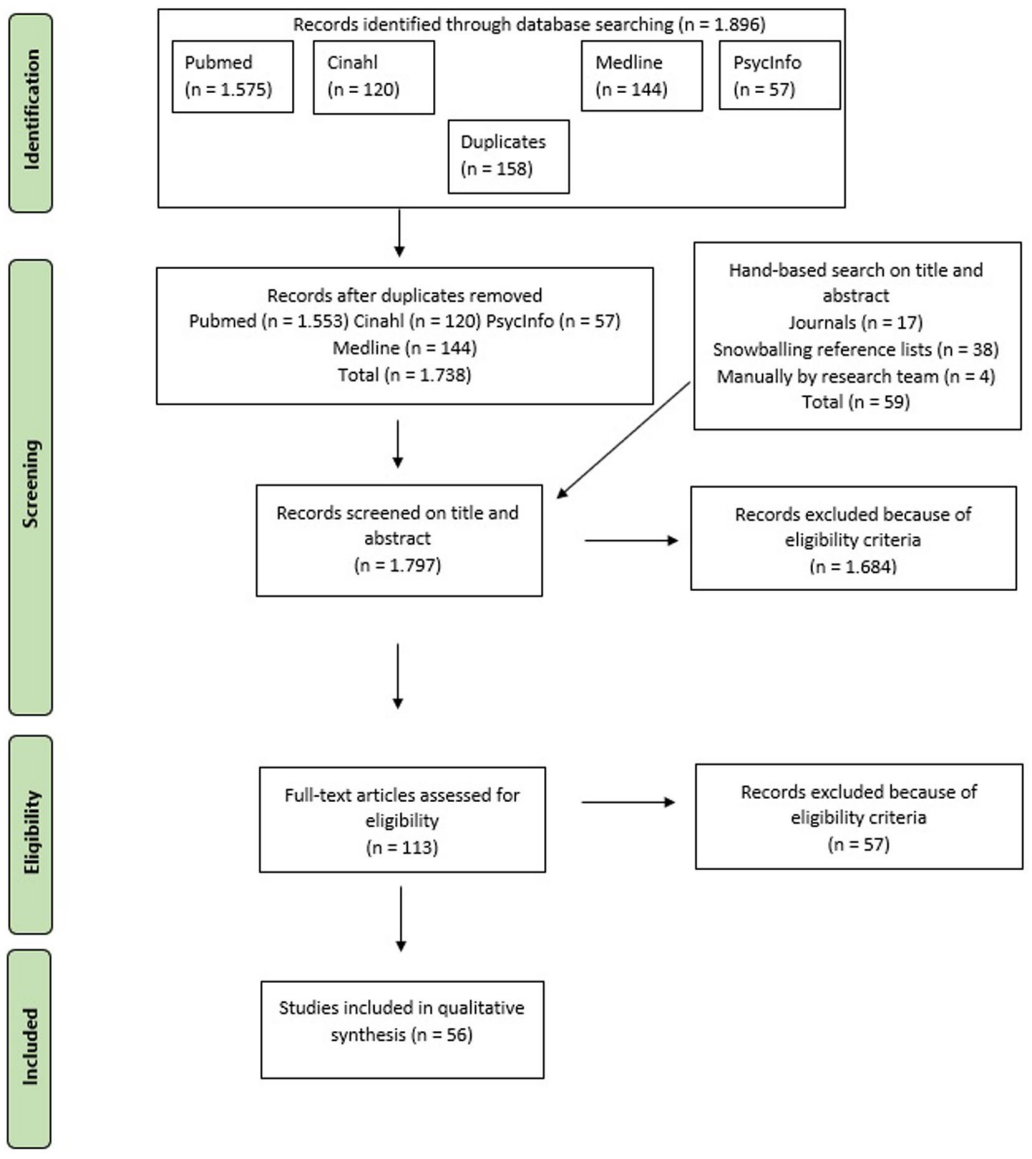
PRISMA flowchart.

After the removal of duplicates and screening all papers on the title, abstract, and full text, the final sample consisted of 56 papers. Figure 2 shows the PRISMA flow diagram of the search.

The 56 papers were published spread throughout our time frame, but by far the most papers were conducted in 2018 (*n* = 8), 2020 (*n* = 7), and 2021 (*n* = 9). Most of the papers were conducted in the United States (*n* = 17). Twenty-seven of the included papers were conducted primarily in Europe; the majority in the United Kingdom (*n* = 7), Netherlands (*n* = 5), and Sweden (*n* = 5). Four papers compared the situations in two countries: Australia and England, England and Italy, England and North Macedonia, and Canada and the United States. Other regions are Canada (*n* = 3) and Australia (*n* = 3). Four papers did not report their country, because they did not focus their research specifically on a country. We included 12 reviews, including three scoping reviews and four systematic reviews. In addition, we included 19 quantitative papers, including five RCTs and seven papers with a longitudinal design. We included 20 papers with a qualitative design, of which six were evaluative papers, seven opinion papers and seven descriptive papers. Finally, we included three mixed-methods studies and five expert papers. Table 1 shows the main information from the 56 papers found.

**Table 1:** Summary of papers included in the scoping review.

Authors (year), Country	Aim of the study	Type of study	Main findings
1. Shattell et al. (2011), United States	Exploring how Assertive Community Treatment (ACT) teams address the physical health needs of people with SMI and learning more about their challenges to addressing the physical health needs of consumers	Qualitative, opinion study, using focus groups (*n* = 33)	Three themes emerged from the analyses: (a) all the ACT teams recognized serious and chronic physical health problems among the consumers they served, (b) ACT teams took on a variety of roles to address the physical health problems of their consumers, and (c) there were several challenges to integrating primary and mental health care within an ACT setting
2. Belling et al. (2011), England	To identify and explore facilitators and barriers perceived to influence continuity of care by health and social care professionals working in and closely associated with Community Mental Health Teams	Qualitative, opinion study, using semi-structured interviews (*n* = 113)	Team leadership, decision-making, and experiences of teamwork support were facilitators for cross-boundary and team continuity; face-to-face communication between teams, managers, general practitioners, and the voluntary sector were facilitators for information continuity. Relational, personal, and longitudinal continuity were facilitated in some local areas by workforce stability. Barriers to cross-boundary and team continuity were specific leadership styles and models of decision-making, blurred professional role boundaries, generic working, and lack of training for role development. Barriers to relational, personal, and longitudinal continuity were created by inadequate staffing levels, high caseloads, and administrative duties that could limit time spent with users
3. Wright-Berryman, McGuire and Salyers (2011), United States	Review the extant literature of consumers as service providers on case management teams and provide an analysis of the impact of consumer providers (CP’s) on consumer outcomes	Review of quantitative empirical studies (*n* = 16)	In their review of the extant literature on CPs on ACT and other case management teams, we found underwhelming evidence regarding clinical outcomes, such as hospitalizations and symptoms
4. Harvey et al. (2011), Australia and England	To investigate differences in the implementation of ACT in Melbourne and London in terms of team structure, staffing and processes, staff experiences, and client characteristics	Quantitative cross-sectional study, using surveys (*n* = 24)	Client characteristics, staff satisfaction, and burnout were very similar. Three of four Melbourne teams made over 70% of client contacts “*in vivo*” compared to only one-third of comparable London teams, although all teams were rated as “ACT-like”. Melbourne teams scored more highly on a team approach. Three-quarters of clients were admitted in the preceding 2 years but Melbourne clients had shorter stays
5. Nordén, Malm and Norlander (2012), Sweden	To explore the effectiveness of the method Resource Group Assertive Community Treatment (RACT) for clients with psychiatric diagnoses	Meta-analysis (*n* = 17)	The meta-analysis concluded that the treatment of clients with Resource Group Assertive Community Treatment yields positive effects for clients with psychoses and that the method may be of use for clients within the entire psychiatric spectrum
6. Nordén et al. (2012), Sweden	The current investigation aimed to qualitatively study the Resource group Assertive Community Treatment (RACT) from the perspective of the trainee case managers	Qualitative opinion study, using case studies (*n* = 19)	Seven index categories emerged, each one of which consisted of several sub-categories: (a) the client; (b) the case manager; (c) the resource group; (d) medical care (e) societal contributions; (f) relatives; and (g) the RACT program
7. Mezzina (2012), Italy	To give an overview of The Trieste Model	A qualitative, descriptive study	This approach is based on five cornerstones: (1) individualize care plans through active negotiation, (2) ensuring comprehensive responsibility of CMHC in all phases of treatment, (3) working on the environment and the social fabric, (4) supporting individual strengths *in vivo*, and (5) fostering service accountability toward the community
8. Firn et al. (2012), England	To demonstrate non-inferiority in clinical effectiveness and thereby show cost efficiencies associated with Flexible Assertive Community Treatment (FACT)	A quantitative longitudinal study, using data from the electronic patient record system (*n* = 112)	The results show AO patients (*n* = 112) transferred to standard CMHTs with FACT had significantly fewer admissions and a halving of bed use (21 fewer admission and 2,394 fewer occupied bed days) while receiving a less intensive service (2,979 fewer contacts). This was offset by significantly poorer engagement but not by increased use of crisis and home treatment services
9. Lee et al. (2012), Australia	Identification of relevant collaborative care models between clinical and non-clinical services to address comorbidities affecting the following domains: homelessness, substance addiction, physical ill-health, unemployment, and forensic issues	Scoping review, supplementing with stakeholder consultation	Governmental and organizational promotion of incentives for cross-sector collaboration is needed. Additionally, with education for staff about comorbidity and the capacity of cross-sector agencies to work in collaboration to support shared clients. Enhanced communication has been achieved through mechanisms such as the co-location of staff from different agencies to enhance sharing of expertise and interagency continuity of care, shared treatment plans and client records, and shared case review meetings. Promoting a “housing first approach” with cross-sector services collaborating to stabilize housing as the basis for sustained clinical engagement has also been successful
10. Scharf et al. (2013), United States	Describe the characteristics and early implementation experiences of community behavioral health agencies that received Primary and Behavioral Health Care Integration (PBHCI) grants to integrate primary care into programs for adults with SMI	Mixed-methods cross-sectional study using semi-structured interviews (*n* = 56)	Barriers to program implementation at start-up included difficulty recruiting and retaining qualified staff. Also issues related to data collection and use of electronic health records, licensing and approvals, and physical space were barriers. By the end of the first year, some problems, such as space issues, were largely resolved, but other issues, including problems with staffing and data collection, remained. New challenges, such as patient recruitment, had emerged
11. Zubritsky et al. (2013), United States	To evaluate the effectiveness of a “Continuum of Care Program” (CCCP) for persons with serious mental health conditions in reducing inpatient use and building a continuum of integrated care that enhanced employment and residential stability	A quantitative, longitudinal study, using a prospective observational design (*n* = 1,154)	Statistically significant changes were seen over 1 year in all outcomes. Housing, employment, and mental health improved, whereas inpatient utilization and level of care need increased. Older individuals receiving higher levels of care at baseline. Those with higher case management and medical service utilization reported higher inpatient use
12. Thornicroft and Tansella (2014), England and Italy	To put community psychiatry into a longitudinal and global perspective and to try to look into the future	Expert opinion	Nine proposals: 1. Central and regional governments should measure the treated percentage of people with mental illness 2. Develop and evaluate new methods to reduce lower life expectance 3. Specific modules to reduce stigma 4. Provide care that service users find accessible and acceptable 5. Careful balance of hospital and community care 6. Invest in treatments that are known to be effective 7. Improve shared decision-making 8. See each other as partners in an integrated system 9. Develop programs for recovery
13. Van Vugt et al. (2014), Netherlands	This study examined the associations between substance abuse problems in severely mentally ill patients, outcome, and Assertive Community Treatment (ACT) model fidelity	A quantitative, longitudinal 2-year follow-up study (*n* = 530)	Patients with an addiction problem had more serious psychosocial problems at baseline. The study indicates that investment by teams to improve a patient’s psychosocial situation can lead to improvements in substance problems
14. Young et al. (2014), United States	This study evaluated the effectiveness of an ACT team that delivered integrated care consistent with integrated dual disorders treatment principles	A quantitative, longitudinal 6-month follow-up study (*n* = 60)	Participants reported statistically significant improvements in mental health symptomatology and residential stability over time. Although there were no changes in substance use. Findings support the effectiveness of the intervention for improving mental health and housing stability among adults with complex behavioral health and housing needs
15. Malm, Ivarsson and Allebeck (2014), Sweden	To evaluate the durability the efficacy of the Integrated Care Program in a Swedish context	RCT	At the 5-year follow-up, significant improvements were noted in social functioning and consumer satisfaction in the IC group (*N* = 35) compared with the RR group (*N* = 31)
16. Malm et al. (2015), Sweden	The efficacy of the RACT program for functioning and user satisfaction in patients with schizophrenic disorders	Review	There were significant improvements in the primary outcome measure for the changes from baseline over 2 and 5 years favoring the Resource group ACT (RACT group). The 5-year findings for primary outcomes were improved social functioning and satisfaction with care for the RACT group
17. Ridente and Mezzina (2016), Italy	To describe the practical and conceptual shift from residential facilities to personalized supported housing processes and to investigate the consequences and effects of the personalized approach	A qualitative, descriptive study	The Individual Health Budget Method boosted the process of more independent and autonomous housing solutions for people with SMI. It seems crucial that community services organize their actions to promote, value, and actively support the ability to empower and involve users, their families, and NGOs. This as an expression of social participation stemming from local communities
18. Rogers et al. (2016), United States	Examine a model for integrating primary care into a community mental health setting	RCT	Participants who engaged with the nurse practitioner experienced gains in perceptions of primary care quality. Health benefits accrued for individuals receiving nurse practitioner services in a mental health setting to address primary care needs. Having a nurse practitioner employed by and stationed in a community mental health setting. and working alongside mental health providers may be a viable way to promote access to and integration of primary mental healthcare
19. Razzaque and Stockmann (2016), England	This article offers an introduction to Peer-Supported Open Dialogue	A qualitative, descriptive study	Peer-supported open dialogue is a variant of the “open dialogue” approach that is currently practiced in Finland. The core principle of the approach is the provision of care at the social network level, Provided by staff who have been trained in family, systems, and related approaches. This staff includes peer workers, who will help to enhance the democratic nature of the meetings around which care is centered, as well as enable such meetings to occur where networks are fragmented or lacking
20. Asad and Chreim (2016), Canada	Explores how peer support providers’ roles are defined and integrated into inter-professional mental health care teams, and how these providers relate to other practitioners and clients	Qualitative opinion study, using semi-structured interviews (*n* = 12)	The findings indicate that: peer support providers experience ambiguity and that some ambiguity may offer benefits; peer support providers enhance team acceptance of their role through several means and strategies; setting boundaries with clients is a delicate issue that requires several considerations that we discuss
21. DiClemente et al. (2016), United States	Describe the Recovery-oriented system of Care (ROSC) framework	A qualitative, descriptive study	Recovery-oriented system of care (ROSC) is a framework designed to address the multidimensional nature of recovery. They created a system for coordinating multiple systems, services, and supports that are person-centered and build on the strengths and resiliencies of individuals, families, and communities
22. Nugter et al. (2016), Netherlands	To investigate social and clinical outcomes and use of care during and after the implementation of FACT	A quantitative, longitudinal study (*n* = 391)	Statistically significant improvements were found in compliance, unmet needs, and quality of life. Improvement of quality of life and functioning was related to the duration of FACT. The percentage of remissions increased by 9%. The number of admissions, admission days, and face-to-face contacts differed between FACT and non-FACT patients but generally decreased
23. Meyer-Kalos et al. (2017), United States	To understand more clearly ACT teams’ field-tested strategies and recommendations for delivering integrated care (physical and mental health self-management for people with SMI)	Qualitative, opinion study, using focus groups (*n* = 16)	Findings from inductive analyses revealed six overarching themes: (1) collaboration with primary care, (2) improvements in engagement, (3) team-focused roles, (4) education and training, (5) recommendations for system-level barriers, and (6) systems collaboration
24. Mueller-Stierlin (2017), Germany	Presenting the results of a quasi-experimental prospective trial comparing the effects of integrated mental health care according to the criteria of the Network for Mental Health (NWpG-IC) to usual mental health care (TAU) in five German regions over 18 months	Prospective, parallel-group controlled multi-center trial (*n* = 511)	NWpG-IC (*n* = 260) and TAU groups (*n* = 251) did not differ concerning most primary and secondary outcomes in our participating patients. However, a significant improvement in terms of patients’ satisfaction with psychiatric care and their perception of treatment participation in favor of the NWpG-IC group was found
25. Milbourn, McNamara and Buchanan (2017), Australia	To investigate the well-being of adults diagnosed with SMI and receiving ACT by applying the occupational well-being framework to the everyday activities of this vulnerable group of people	Qualitative evaluative study, using semi-structured interviews (*n* = 11)	Participants’ everyday activities and occupational well-being appeared severely restricted and largely determined by the type of care they received. There was minimal evidence of the well-being descriptors, though all the participants reported experiencing some form of pleasure, even though some of the pleasurable experiences negatively impacted their health
26. Svensson, Hansson and Lexén (2018), Sweden	To explore the extent to which clients assigned to the FACT board for ACT intensive care stabilized with improved everyday functioning, social outcomes, and changes in healthcare use	A quantitative longitudinal study, using quasi-experimental naturalistic observational design (*n* = 93)	There was a significant positive change in everyday functioning and the SIX-item “friendship” at the 18-month follow-up. A positive correlation was also found between everyday functioning and the SIX-item “friendship” and a negative correlation between the duration of ACT and everyday functioning. A significant increase in the number of inpatient hospital days and psychiatric outpatient visits also occurred
27. Gupta et al. (2018), England	Describe how primary care practitioners can help stimulate such a grand alliance for health, by operating at four different levels – as individual practitioners, as organizations, as geographic clusters of organizations, and as policymakers	Expert paper	1. Use initiatives like New Care Models and Primary Care Home to work with local general practices, public health, social care, mental health services, voluntary groups, and others to align plans for positive mental health in the locality. 2. Work in clusters of practices and their extended primary teams to integrate mental health care into route care. 3. Write live manuals, tailored to local needs, to inform practitioner decisions, self-help, and care pathways. 4. Develop multidisciplinary teams 5. Highlight wider determinants of mental illness 6. Consider mental health needs of those who have long-term conditions 7. Signpost ways for people to self-care and make useful contributions to society
28. Huck et al. (2018), United States	To identify strategies for encouraging greater levels of physical activity among community-dwelling individuals living with SMI participating in assertive community treatment	Qualitative opinion study, using semi-structured interviews (*n =* 19)	Participants identified a variety of strategies used by their ACT provider to encourage physical activity, such as group physical activities and incentive programs. Several recommendations related to skills training, intervention characteristics, and motivational strategies were identified by the participants as well
29. Woody et al. (2018), Australia	Describe or evaluate characteristics or practices for multidisciplinary team reviews of patients with SMI to discover: (1) what are the characteristics and practices of MDTRs in mental health and (2) what modifications should be made for patients with complex clinical needs?	Systematic Review	Important characteristics and practices identified included routine monitoring and evaluation, good communication, equality between team members, and clear documentation practices. Success factors included defined leadership and clear team goals. Four sources described considerations for patients with complex clinical needs, including allocating sufficient time for discussion, maintaining connections with community providers, and ensuring culturally sensitive practices
30. LeFebvre et al. (2018), Canada and United States	We conducted an exploratory study to collect data on transition rates, community services outside of ACT, the barriers to transition, and the strategies to overcome these barriers among ACT teams serving clients in urban and rural areas	Mixed-methods study, using semi-structured interviews (*n* = 8)	On average, teams transitioned about 6% of their clients over our 3-year study period. Urban and rural teams described both similar and distinct clinical and systemic barriers, such as client reluctance to transition and finding psychiatric follow-up outside of ACT
31. Killaspy et al. (2018), Europe	Present a summary of what is known so far on the most effective Approaches in Community Mental Health Care	Expert opinion	The highest level of deinstitutionalization development has been seen in the inclusion of inpatient psychiatric units in general hospitals. Followed by the development of outpatient services in general hospitals and the community, daycare services, and community mental health centers. Principles: 1. Protection of human rights 2. Accessibility and equity 3. Recovery 4. Care in the community 5. Coordination and integration of care 6. Community participation of users and families
32. Dalton-Locke et al. (2018), England	Identify potential service characteristics that were associated with quality of care with the QuIRC-SA in mental health supported accommodation services in England	Quantitative, cross-sectional study (*n* = 150)	The local authority area in which the service is located, the service size (number of beds/places), and the usually expected length of stay were each negatively associated with up to six of the seven QuIRC-SA domains. Staffing intensity was positively associated with two domains (Therapeutic Environment and Treatments and Interventions) and negatively associated with one (Human Rights)
33. McPherson, Krotofil and Killaspy (2018), England	Synthesize the current evidence on mental health and psychosocial outcomes for individuals residing in mental health supported accommodation services	Systematic Review (*n* = 82)	Supported accommodation is effective across a range of psychosocial outcomes, reducing hospitalization rates and improving appropriate service use
34. Farkas and Coe (2019), United States	Describe the evolution of supportive housing and its basic tenets, identifying the challenges and some efforts to address them	Review	Supportive housing emerged as a model based on integrated, permanent, affordable housing. Housing is selected by the person, with flexible supports that are functionally separate, but available as needed and wanted. Current challenges confront the sustainability of supportive housing, with some efforts being made by housing groups to address these challenges
35. Rochefort (2019), United States	Giving an overview of the history of ACT	Review	Several factors have functioned to fuel and constrain ACT diffusion. The former category includes policy learning through research; the role of policy entrepreneurs; ACT’s acceptance as a normative standard; and a thriving international epistemic community. The latter category includes cost concerns, fidelity demands, shifting norms, research contradictions and gaps, and a multifactorial context affecting program adoption. Currently, the program stands at a crossroads, strained by the principle of adherence to a long-standing operational framework, on the one hand. On the other hand, calls to adjust to an environment of changing demands and opportunities
36. Keet et al. (2019), Europe	Contribute to the discussion on how to improve structures in mental healthcare and narrow the gap between evidence, policy, and practice in Europe	Expert opinion	High-quality community-based mental health care should conform to six principles: 1. Human Rights 2. Public Health 3. Recovery 4. Effectiveness of interventions 5. Community Network of Care 6. Peer Expertise
37. Gaebel et al. (2020), Germany	To update recommendations regarding care coordination across different mental healthcare services	Systematic meta-review (*n* = 23)	The studies compare either several components of coordinated care models or focus on one specific coordinated care component: (a) Case management, integrated mental health services, and home treatment, (b) Crisis intervention services, (c) Transition from inpatient to outpatient care and vice versa, return to work, (d) Integrating general and mental healthcare, (e) Technology and self-management in care coordination, and (f) Quality indicators and economic evaluation
38. Davidsen et al. (2020), Denmark	To explore different professionals’ and patients’ experiences of trans-sectoral collaboration for patients with SMI and concurrent physical disease within the Danish health and social care system	A qualitative evaluative study, using interviews, observation, focus groups, workshops, and notes from meetings	Professionals in general practice and social psychiatry felt that they were left with the responsibility for actions taken by hospital psychiatry without the opportunity to discuss their concerns with psychiatrists. There were also cultural differences between health care and social psychiatry, expressed in ideology and language. Social psychiatry had an existential approach to recovery, whereas the views of health professionals were linked to symptom control and based on outcomes
39. Wusinich et al. (2020), United States	To explore the perspectives of individuals enrolled in Parachute	A qualitative evaluative study using interviews (*n* = 18)	Participants reported that they valued the accessibility and flexibility of Parachute as well as their relationships with, and the lack of hierarchy within, the Parachute team. Responses to the structure of network meetings and Parachute’s approach to medication were mixed. A few participants struggling with what they felt was a lack of urgency and others experiencing the approach as holistic. Many enrollees and network members reported that Parachute improved their self-understanding and relationships with each other
40. Zomer et al. (2020), Netherlands	Presenting the key characteristics of the ART model	A qualitative, descriptive study	The ART model combines an active role for professionals, service users, and significant others, with a focus on recovery and cooperation between service users, family, and professionals in the triad. The principles of ART are translated into seven crucial steps in care and a model fidelity scale to provide practical guidelines for teams implementing the ART model in practice. The ART model guides tailored recovery-oriented care and support to this “low-volume high-need” group of service users in long-term mental health care. The aim is to alter their perspective and take steps in the recovery process
41. Hirdes et al. (2020), Worldwide	An overview of the RAI suite of mental health instruments, which is designed to function as an integrated assessment and screening system to provide a holistic view of the person’s strengths, preferences, and needs	A qualitative, descriptive study	The instruments form an integrated mental health information system. They share a common assessment language, conceptual basis, clinical emphasis, data collection approach, data elements, and care planning protocols
42. Bajraktarov, Kalpak and Jovanovic (2020), North Macedonia and England	To identify and map the available evidence on recent innovations in community mental healthcare across the globe	Scoping Review (*n* = 35)	A growing body of evidence shows that integrative care is the new standard of care for people with mental illnesses. Continuity of care from the emergency department to community mental health services is a necessity. Key approaches found in the reviewed studies include collaborative care with the inclusion of peer workers, growing use of e-health and telepsychiatry, improved reforms on national mental health policies and de-institutionalization, modification of outreach models and mental health promotion in the community
43. McGinty et al. (2021), United States	To discuss the current state of the evidence on integrated care models based on the specialty mental health system and to identify priorities for future research, policy, and practice	Expert opinion	Key research priorities include identifying the active ingredients in multicomponent integrated care models and developing and validating integration performance metrics. Key policy and practice recommendations include developing new financing mechanisms and implementing strategies to build workforce and data capacity. Forum participants also highlighted an overarching need to address socioeconomic risks contributing to excess mortality among adults with serious mental illness
44. Brar et al. (2021), United States	This study focused on understanding the effectiveness of a 12-month Learning Collaborative in scaling a BHH strategy. More specifically, we were interested in understanding if a BHH could impact physical health outcomes including tobacco use and hypertension	A quantitative, longitudinal study using observational process evaluation	Providers reported increases in screening rates and wellness goals related to tobacco use and hypertension. Also reductions in tobacco use and blood pressure readings among participating individuals were reported. Evidence presented indicates that a Learning Collaborative of community mental health providers is a feasible quality improvement approach to scale integration of physical and behavioral health care for individuals with serious mental illness
45. Tjaden et al. (2021), Netherlands	To determine whether using Resource Groups (RGs) within Flexible Assertive Community Treatment (FACT) has favorable effects on empowerment and recovery-related outcomes in people with SMI	RCT	These findings show that working with RGs improves empowerment and other mental health outcomes in people with SMI who receive community-based mental health services. This method of network-oriented care empowers people with SMI within their environment
46. Marshall et al. (2021), Canada	To develop and refine a framework to guide occupational therapy practice and research in homelessness	Qualitative opinion study, using surveys (*n* = 17)	Stakeholder feedback was categorized into eight recommendations: (1) Revision to the “four processes”; (2) Emphasizing social justice and systems-level advocacy; (3) Reflecting intersectionality; (4) Emphasizing meaningful activity; (5) Emphasizing peer support; (6) Incorporating a focus on independent living skills; (7) Increasing a focus on an activity for addressing substance misuse; and (8) Acknowledging cognitive and physical health
47. Gaiser et al. (2021), United States	To better define the roles of peers and their unique contributions to behavioral health care	Systematic Review (*n* = 23)	Peers were employed most frequently in mental healthcare roles in the Department of Veterans Affairs, hospitals, and community health facilities. A total of 14 studies observed significant clinical improvements in participants’ social functioning, quality of life, patient activation, and behavioral health
48. Brekke et al. (2021), Norway	To explore and describe service user experiences of how receiving services from a FACT team may support or inhibit citizenship	A qualitative evaluative study using interviews (*n* = 32)	The findings showed that FACT may support citizenship by relating to service users as whole people, facilitating empowerment and involvement, and providing practical and accessible help
49. Lama, Fu and Davis (2021), Canada	To initiate a discussion of the ideal occupational therapy practice from the perspective of occupational therapists working on ACT teams	Qualitative descriptive study, using semi-structured interviews (*n* = 11)	Three themes emerged: (a) Engaging in practice “with intention”; (b) Finding the space for occupational therapy practice, and (c) Supporting clients in their recovery to find their best occupational self
50. Trane et al. (2021), Norway	To explore how FACT teams are integrated into the existing formal public service system, how they function and affect the system, and describe some influencing factors of this	A qualitative evaluative study, using focus groups (*n* = 40)	The analysis revealed five main themes regarding FACT teams: (1) They form a bridge between different services; (2) They collaborate with other services; (3) They undertake responsibility and reassure other services; (4) They do not close all gaps in service systems; and (5) They are part of a service system that hampers their functioning
51. Nielsen et al. (2021), Denmark	To evaluate the effect of FACT on mental health care outcomes compared with treatment from standard community mental health teams (CMHTs) or assertive community treatment (ACT) teams in Denmark	Quasi-experimental controlled study	The number of outpatient contacts was higher for patients receiving FACT than for those in the control groups. Patients receiving FACT had fewer admissions than those in the control groups. However, there were no significant differences in total inpatient days, use of coercion, episodes of self-harm, or deaths
52. Ciampa, Roca and Lysaght (2022), Canada	Aims to explore what supports are effective in moving service users toward work integration	Quantitative, cross-sectional study, using surveys (*n* = 145)	The majority of respondents (64,8%) were not employed. Those who were working presented higher levels of functional capacity than those who were not working. The majority of participants reported not receiving supports toward support for work integration
53. Edmundson et al. (2022), United Kingdom	To understand what healthy means to people with SMI and the barriers and facilitators to living a healthy lifestyle	Qualitative, opinion study, using focus groups	Five themes were identified: (1) mental health is the main priority. And the other themes were barriers to a healthy lifestyle, represented as (2) a vicious cycle, and three themes, which were facilitators – (3) the importance of place, (4) meaningful activities, and (5) the importance of others
54. Fisher et al. (2022), United States	To bring the voice of the consumer with SMI to assist with the integration of primary care and mental health services	A mixed-method study, using focus groups and surveys	Three relevant themes emerged: primary care experiences; health care stigma; and social determinants as barriers to health. Individuals with SMI supported the integration of care, with careful consideration given to social determinants of health, patient privacy, and respect between providers and patients
55. McCormick et al. (2022), United States	To determine if environmental novelty was associated with neurocognitive function among adults with SMI	Quantitative, cross-sectional study, using GPS	Homebodies demonstrated significantly poorer cognitive function than venturers. This relationship was not mediated by several unique destinations or breadth of community participation activities
56. Meyer et al. (2022), worldwide	Synthesize social network research in Clubhouse members and results from this review may improve understanding of the role of supportive relationships in mental illness recovery	Scoping review	Overall findings suggest that network size is not consistently associated with reported loneliness, social support, recovery, or quality of life. A deep relationship with at least one supportive person, level of perceived affiliation with Clubhouses, or positive comments from network members may be more or equally valuable than a larger network

### 3.2. Thematic analysis

We found ingredients of community-based mental healthcare for persons with SMI in 12 areas: 1. multidisciplinary teams; 2. collaboration within and outside the organization; 3. attention to several aspects of health; 4. supporting full citizenship; 5. attention to the recovery of daily life; 6. collaboration with the social network; 7. tailored support; 8. well-trained staff; 9. using digital technologies; 10. housing and living environment; 11. sustainable policies and funding; and 12. reciprocity in relationships. The subcategories were indicated in the results in bold. Table 2 shows which ingredients were found in which papers, arranged by study design. All papers were classified into nine categories of study designs. The first category contains all types of reviews, including one systematic meta-analysis. The quantitative papers were divided into three categories: RCTs, cross-sectional, and longitudinal. The qualitative papers were also divided into three categories: evaluative (papers in which respondents shared their experiences with the researchers); opinion (in which participants are asked for their opinions about a phenomenon); and descriptive (papers describing a phenomenon). The remaining two categories are mixed-method and expert papers (papers without empirical research but with the opinion of the authors).

**Table 2:** Results of the thematic analysis.

Category of study design	Quantitative					Qualitative				No. papers per area
	1. Review	2. RCT	3. Cross-sectional	4. Longitudinal	5. Mixed-method	6. Evaluative	7. Opinion	8. Descriptive	9. Expert paper	
Area of ingredients										
1. Multidisciplinary teams	(29–33)	(34–36)	(37)	(38–42)	(43)	(44, 45)	(46–48)	(49–51)	(52, 53)	25
2. Collaboration within and outside the organization	(29, 31, 54)			(40, 55, 56)	(57, 58)	(45, 59, 60)	(46, 48, 61)	(49, 62)	(3, 52, 53, 63)	20
3. Attention to several aspects of health	(31)		(64)	(41, 55)	(57, 58)		(46, 48, 65–67)	(62, 68, 69)	(52, 53, 63, 70)	18
4. Supporting full citizenship	(29, 71, 72)	(73, 74)				(60, 75)	(48)	(62, 69)	(3, 52, 53, 63)	14
5. Attention to the recovery of daily life			(76)				(48)	(49, 50, 62, 68, 69, 77)	(3, 52, 53, 63)	10
6. Collaboration with the social network	(71, 72, 78)	(73, 74)				(75)		(49, 51, 68)	(3)	10
7. Tailored support	(79)					(60, 80)		(49, 68, 77)	(3, 63)	8
8. Well-trained staff	(54, 81)		(82)		(58)			(49, 51)	(53, 63)	8
9. Using digital technologies	(29, 31, 81)				(58)			(49)	(52, 63)	7
10. Housing and living environment	(24, 79)		(82)					(77)	(52)	5
11. Sustainable policies and funding	(29)							(49)	(53, 70)	4
12. Reciprocity in relationships							(46)	(68)	(52)	3
Total papers (*N* = 56)	12	5	4	7	3	6	7	7	5	

#### 3.2.1. Multidisciplinary teams

Multidisciplinary teams came up as important in twenty-five of the included papers. Five were reviews, three were RCTs, one was a quantitative cross-sectional paper, five were quantitative longitudinal papers and one a mixed-method paper. Additionally, two were qualitative evaluative papers, three were qualitative opinion papers, three were qualitative descriptive papers and two were expert papers.

Five papers recommend close involvement within different disciplines in multidisciplinary teams. Of these, three were qualitative papers (46, 47, 49), one expert paper (52), and one review (29). Therefore, one RCT finds positive results with regard to the health benefits for individuals for having received nurse practitioner services in a mental health setting to address primary care needs (34). In addition, two papers with a qualitative design emphasize adding an occupational therapist to a multidisciplinary team (44, 50). Finally, seven papers show the value of peer support to multidisciplinary teams; of which these seven papers, there are three reviews (29–31), three qualitative papers (47, 48, 51), and one expert paper (53).

An example of working in multidisciplinary teams is the (flexible) assertive community treatment ((F)ACT) teams. We found mainly empirical studies about the implementation and efficacy of (F)ACT and collaboration with (F)ACT teams. Of these, we found two reviews (32, 33), one RCT with positive results (35), and one RCT without significant results (36). In addition, six quantitative papers (37–42), one mixed-method paper (43), and two qualitative papers (44, 45) report on (F)ACT. Lastly, we found one review about the history of assertive community treatment (ACT) (32).

#### 3.2.2. Collaboration within and outside the organization

Collaboration inside and outside the mental healthcare organization was studied in 20 of the included papers. Three were reviews, two were qualitative longitudinal studies, one was a quantitative longitudinal paper, three were qualitative evaluative papers, three were qualitative opinion papers, two were qualitative descriptive papers, two were mixed-method papers, and four were expert papers.

Intersectoral collaboration is often mentioned in the literature found. The collaboration between mental healthcare, physical care, and social service sectors was found in four qualitative papers (45, 46, 48, 49) and in one scoping review (54). In addition, collaboration between the government and the mental health sector was found in three qualitative studies (45, 59, 60) and one quantitative paper (40). Furthermore, we found three expert papers about collaboration in an integrated care system (3, 53, 63). Therefore, one systematic metareview (31), one mixed-method paper (57), one quantitative study (55), and one opinion paper (52) recommend an integrated care system with the integration of primary care in mental healthcare, and one descriptive paper shows an integrated mental health information system (62).

Growing evidence shows that integrative care is the new standard of care for people with mental illnesses, with the necessity of continuity of care from the emergency department to community mental health services. Continuity of care was found in two scoping reviews (29, 54), one quantitative longitudinal paper (56), and one qualitative paper (61).

Finally, two qualitative papers (59, 61) and one mixed-method paper (58) show the facilitators and barriers to intensive, intersectoral collaboration in community mental healthcare, such as cultural differences between the sectors as a barrier and face-to-face communication as a facilitator.

#### 3.2.3. Attention to several aspects of health

Several aspects of health were studied in 18 of the included papers. Of these 15 papers, one was a systematic-meta review, two were quantitative longitudinal papers, one was a cross-sectional paper, eight were qualitative opinion papers, two were mixed-method papers, and four were expert papers.

Eight papers mention the focus on physical health in mental healthcare. Three qualitative, one mixed-method, and one quantitative paper report on the integration of physical health in mental healthcare (46, 55, 57, 65, 66). According to one expert paper, healthcare services need to recognize the far lower life expectancy among people with mental disorders and develop and evaluate new methods to reduce this health disparity (63). Respondents of a qualitative, opinion study stated that their main preoccupation and motivation was to be mentally well but they also recognized that many things that improve physical health also improved their mental health (67). In addition, one paper with a mixed-method design found that there is growing interest in models integrating physical healthcare delivery, management, or coordination into specialty mental health programs in the United States (58). One expert paper indicates the same (70). Finally, one systematic review found that this integration improves rates of immunization and screening for medical disorders, accompanied by positive effects on physical health, as well as improving general medical outcomes (31).

Another important aspect of health is cognitive functioning, which was found in two papers. One qualitative study mentioned that cognitive and physical health conditions might impact individuals’ ability to function in their daily lives during and after homelessness (48). In addition, one cross-sectional paper found that homebodies reported significantly poorer cognitive function than venturers (64).

In addition, we found two papers on psychosocial health. One quantitative study indicates that investment by teams to improve a patient’s psychosocial situation can lead to improvements in substance problems (41) and one expert paper aligns the importance of addressing social determinants of health within integrated care models for people with SMI (70).

Also, three qualitative papers (62, 68, 69) and one expert paper (52) emphasize the importance of positive health. The focus in positive healthis on the strengths, preferences, needs, and wishes of the service user, families, and communities that contribute to recovery.

Finally, two expert papers emphasize public health as actions seek to achieve equity between groups and a state of population-level health (52, 53).One expert paper shows the role of mental and public health promotion and prevention, taking the needs of the entire population into account (53).

#### 3.2.4. Promoting full citizenship

Promoting full citizenship was a topic of relevance in 14 of the included papers. Three were reviews, two were RCTs, two were qualitative evaluative papers, one was a qualitative opinion paper, two were qualitative descriptive papers, and four were expert papers.

Human rights and destigmatization contribute to promoting full citizenship and are addressed in six papers (3, 29, 48, 53, 63, 82). One quantitative paper found that staffing intensity was negatively associated with human rights (82). One expert paper reports that historically the protection of human rights is one of the drivers for deinstitutionalization. Additionally, people with SMI experience more violations than others and suffer from stigma and discrimination (53). With this in mind, another expert paper states that mental health services should provide specific modules to reduce stigma and discrimination experienced by people with SMI (63). The same paper also states that some programs to reduce stigma and discrimination are presently active at the local level. They now need to be coordinated at the national level and adequately financed (63). In addition, providing training and coaching to health and social care staff on recovery and rights can reduce human rights violations that occur in the context of mental health services (53). One scoping review (29) found that there were an overwhelming number of anti-stigma campaigns from 1995 to 2015, but with a lowering trend of publication year over year on this topic.

In addition, several papers describe the Resource Group methodology that also promotes citizenship because the main feature of this methodology is that ownership and direction lie with the client. Of the included studies, there were two RCTs (73, 74) with positive results, one meta-analysis (71), one review (72), and one qualitative paper (75). Finally, we found some papers on self-reliance. Two qualitative papers describe that a recovery-oriented system of care should give a holistic view of a person’s strengths and build on the strengths and resiliencies of individuals, families, and communities (62, 69). One qualitative evaluative paper finds that FACT may support citizenship by relating to service users as whole people, facilitating empowerment and involvement (60).One expert paper states that signpost ways are needed for people to self-care, make useful contributions to society (52), and to be able to feel a fully-fledged citizen.

#### 3.2.5. Attention to the recovery of daily life

Recovery of daily life was studied in 12 of the included papers. Of these 10 papers, one was a quantitative cross-sectional paper, one was a qualitative opinion paper, four were qualitative descriptive papers and four were expert papers.

We found several aspects of community-based support that contribute to recovery. According to one qualitative paper (68) and two expert papers (53, 63), mental health services should develop dedicated programs for recovery. Also, three qualitative papers (48, 49, 77) focus on gaining and regaining skills for more independent living *in vivo*. Moreover, two expert papers mention that signpost self-care options (52) and recovery colleges can contribute to the recovery of daily life (3). Besides that, one expert paper states that evidence-based, psychosocial interventions should be deployed to support individuals to achieve both personal recovery and increased independence (3). In addition, one qualitative study reports that occupational therapists should support clients in their recovery to find their best self (50). Lastly, one cross-sectional paper states that most of their respondents (64.8%) were not employed, but those who were working presented higher levels of functional capacity than those who were not (76).

#### 3.2.6. Collaboration with the social network

Collaboration with the social network of the client was studied in 10 of the included papers. Of these 10 papers, three were reviews, two were RCTs, one was a qualitative evaluative paper, three were qualitative descriptive papers and one was an expert paper.

Many papers mention several models in which it is important to involve the clients and their social network in the recovery process. We found the collaboration with the social network papers applying the Resource Group methodology in two RCTs (73, 74), one meta-analysis (71), one review (72), and one qualitative paper (75). In addition, we found two qualitative descriptive papers that described approaches that place collaboration with the social network at the center of the client’s recovery process: namely, the Peer-Supported Open Dialogue (51), and the Active Recovery Triad (ART) model (68). Also, one expert paper and one qualitative paper mention that actively supporting the ability to empower and involve users and their families is important in community mental healthcare (3, 49). Finally, one scoping review suggests that network size is not consistently associated with reported loneliness, social support, recovery, or quality of life. A deep relationship with at least one supportive person may be more or equally valuable than a larger network (78).

#### 3.2.7. Tailored support

Tailored support came up as important in eight of the included papers. Of these eight papers, one was a review, two were qualitative evaluative papers, three were qualitative descriptive papers, and two were expert papers.

Two expert papers and three qualitative papers state that mental healthcare should provide care that service users (and their family members) find accessible and acceptable (3, 60, 63, 80) and 24/7 available (77). In addition, one qualitative paper states that recovery-oriented care should be more effective when combined with support when required (49).One review found that support must also be flexible and user-driven (79). Therefore, one qualitative paper states that care should always be started with a Care Planning Meeting (68) and, according to one expert paper (63) and one qualitative paper (77), individualized care plans should be made through shared-decision making. Also, one expert paper states that care should be independent of location (63). Finally, we found two descriptive papers that describe that deinstitutionalization today means positive risk-taking and serious rethinking of questions in terms of distance, power, and language (77) and it provides new approaches to opportunity and safety (49).

#### 3.2.8. Well-trained staff

Well-trained staff came up as important in eight of the included papers. Of these papers, there were two reviews, one quantitative cross-sectional paper, two qualitative descriptive papers, one mixed-method study, and two expert papers.

Two papers reported on education for mental healthcare staff. One review found that education for staff regarding identifying and responding to comorbidity is important (54).One qualitative descriptive paper states that peer-support open-dialogue teams should be trained in family systems (51). Furthermore, we found two expert papers and one qualitative paper that recommend the knowledge and use of evidence-based interventions and treatment by caregivers to provide social inclusion and recovery (49, 53, 63). In addition, one quantitative paper found that service teams should be of moderate size with adequate staffing to support service users in gaining and regaining skills for more independent living (82).One systematic review identified characteristics of well-trained staff, with practices that included routine monitoring and evaluation, good communication, equality between team members, and clear documentation practices (81). On the other hand, one mixed-method paper found difficulty in recruiting and retaining qualified staff (58).

#### 3.2.9. Using digital technologies

Using digital technologies was studied in seven of the included papers. Of these seven papers, three were reviews, one was a qualitative descriptive paper, one was a mixed-method study, and two were expert papers.

One scoping review (29) found that digital platforms have an important role in improving the reach, scale, and accessibility of community-based support. Additionally, digital platforms add addressing public health issues and peer-led interventions are achieved effectively through the utilization of social media tools (29). Therefore, the same scoping review found that eHealth tools are becoming prevalent in the processes of promotion, prevention, and treatment in mental healthcare. In addition, the increased use of these eHealth tools continues to shape the future of community mental healthcare, particularly in low-access areas and areas where certain local expertise is lacking (29). Also, one expert paper states that the use of digital technologies should encourage self-care (52). Besides that, one mixed-method study and one systematic meta-review recommended the use of digital technology in electronic health records to enhance care coordination and promote integrated care (31, 58). Finally, one systematic review (81), one qualitative paper (63), and one expert paper (49) state that digital monitoring through technology may improve practices and patient outcomes.

#### 3.2.10. Housing and living environment

Housing and living environment came up as important in five of the included papers. Two were reviews, one was a quantitative cross-sectional paper, one was a qualitative descriptive paper, and one was an expert paper.

One review (79) describes the principles of the supportive housing approach in the United States. In addition, one systematic review (24) found positive results with regard to supported accommodation on several outcomes and the importance of connection to, and affiliation with, the living environment.

Furthermore, one expert paper (52) and one quantitative paper (82) state that writing live manuals tailored to local needs helps to stimulate a grand alliance for health. Also, one qualitative paper (77) describes the cornerstones of the Trieste Model. Two of the cornerstones are actively working on the environment and the social fabric, and service accountability toward the community.

#### 3.2.11. Sustainable policies and funding

Sustainable policies and funding came up as important in four of the included papers. Of these four papers, one was a scoping review, one was a qualitative descriptive paper, and two were expert papers.

One expert paper states that the integration of community mental healthcare services, sectors, and collaboration with the social network of the service user can be hindered by a financing system that favors institutional care. Therefore, it is recommended to create a flexible financing system that allows incentives for different services that address the relevant life domains of people with SMI in the community (53). Another expert paper states that financial barriers are also encountered when integrating general practitioner care and mental healthcare (70). In addition, one qualitative paper describes a successful financial model that was developed in Italy. The personal health budget includes all economic, professional, and human resources needed to trigger a process aimed at restoring a person – through an individual rehabilitation process – to an acceptable level of social functioning (49). Finally, one review found that improved reforms on national mental health policies and deinstitutionalization are important for community mental healthcare (29).

#### 3.2.12. Reciprocity in relationships

Reciprocity in relationships is a topic of interest in three of the included papers. Of these three papers, one was a qualitative opinion paper, one was a qualitative descriptive paper, and one was an expert paper.

This topic is about the reciprocity in relationships between clients and caregivers, but also in contributions to society by all people. One qualitative paper shows the importance of establishing and maintaining contact between the caregiver with the service user, by building a mutual relationship of trust (68). Therefore, one expert paper found that all people, with or without mental health problems, should make useful contributions to society, including paid and voluntary work that helps strengthen the local community, appreciate those around them and increase their webs of trusted relationships (52). Finally, to promote reciprocity, one qualitative opinion study states that the use of person-centered strategies is important. This focus on a person’s interests and goals was frequently indicated to foster relationships, gain trust, and develop self-efficacy (46).

## 4. Discussion

### 4.1. Summary of main findings

With this scoping review, we aimed to give a comprehensive overview of existing and upcoming community mental healthcare approaches to discover the current vision explained in areas of ingredients. To our knowledge, there are still a few publications that attempt to combine all the necessary elements for community mental health (53, 83, 84). We found 56 papers that met the inclusion criteria. Thematic analysis resulted in 12 areas of ingredients for community mental healthcare. In this section, we answer our research questions and show what was striking in the found literature. Finally, we present the strengths and limitations of our scoping review and our conclusions.

We aimed to give an overview of the existing and upcoming insights on community mental healthcare for people with SMI. Based on the number of papers found, most attention is paid to several aspects of health, multidisciplinary teams, collaboration within and outside the organization, collaboration with the social network, and supporting full citizenship. However, empirical evidence from quantitative studies was found in only four of the 12 areas based on our included papers: multidisciplinary teams; collaboration with the social network; collaboration within and outside the organization; and supporting full citizenship. Nevertheless, the other areas that are not yet supported by evidence in this scoping review are no less important for community mental healthcare. Although no empirical research has yet been done on these topics, they are being addressed in several papers. This shows that there is increasing attention to them in the field.

Notably, given the low number of included empirical studies from the welfare or supported housing sector, we can conclude that little empirical research has been done on community mental healthcare in these sectors for this target group. The few empirical studies we from the mental health care sector and were primarily about (F)ACT and the Resource Group methodology. Even though (F)ACT has been around for a few decades, this shows that for the last 10 years (F)ACT has remained an important model within community mental healthcare for this target group to achieve recovery. Additionally, given the number of papers reporting on it and the evidence provided, collaborative mental healthcare within teams, organizations, and clients and their social network has been seen as important over the past decade.

In addition to the topics found that received attention for more than 10 years within community mental healthcare, such as recovery, tailored support, and multidisciplinary teams, we found several papers that are about more recent and innovative areas. Such as, reciprocity in relationships; sustainable policy and funding; using digital technologies; and supporting full citizenship. The results from this scoping review show that in recent years new shifts are taking place in the field of mental healthcare, whereby there is more attention paid to full citizenship, but empirical research is still lacking. Further, the more innovative areas were published in more recent literature, but frequently in expert papers. Due to the few RCTs found, we cannot conclude that these areas are also the most important, but we could say that these areas can form the basis for further research in community-based mental healthcare to provide social inclusion and recovery in the future.

Recovery was one of our research terms and we expected recovery to be an important part of our scoping review because more and more studies have been conducted on the areas of recovery. Ten included papers reported on the recovery of daily life, but no empirical studies were found on that topic. This is probably due to the exclusion of intervention studies that focus on a single life domain and did not seek collaboration, which is often the case in studies of recovery and all aspects of health. The upcoming attention to recovery-based care for persons with SMI is also shown in recent scoping reviews. Bitter et al. (85), in their review on recovery interventions for supported housing and clinical settings, found 53 papers, of which about a quarter of recovery interventions showed added value based on RCTs and half of them had initial promising results based on case studies and follow-up designs without a control group. Additionally, van Weeghel and colleagues (86) reviewed the conceptualization of recovery, showing that personal recovery is conceptualized as complementary to clinical recovery and represents processes rather than outcomes. They state that a broad framework of recovery is required, and more research is needed into the working mechanisms of personal recovery processes. Our search and the previous scoping reviews show that recovery is still a guiding concept for people with SMI that needs to be paid attention to both in today’s and future community-based mental healthcare, but more empirical research is necessary to find the working mechanisms that contribute to recovery.

Further, we found many papers concerning multidisciplinary teams. Peer supporters (29, 30, 47, 48, 51, 53), occupational therapists (44, 50), and nurses (34) are frequently mentioned as important disciplines in a multidisciplinary team, besides the regular disciplines of psychologists, psychiatrists, and social psychiatric care workers. Peer supporters have been a part of the (F)ACT teams for more than our searched 10 years (87). Adding peer supporters to multidisciplinary teams is found in seven papers, including three reviews. Because of this, there is much evidence that peer support adds value to multidisciplinary teams.

Additionally, intersectoral collaboration appears regularly in the literature. First, several papers studied the collaboration with primary care to provide the physical health of people with SMI and the importance of attention being paid to clients’ physical health in mental healthcare. This builds on previous research that shows that people with SMI experience premature mortality of around 15–20 years earlier than the general population (88), have a high prevalence of substance use disorder (89), and are at risk of the development of often preventable secondary health conditions (90, 91). Second, collaboration with the municipalities is recommended in the literature. The importance of collaboration between mental healthcare and community services is lacking in the papers. Currently, the mental healthcare sector seems the most important party in the found literature to support this target group. The collaboration between mental healthcare and municipal services is most often mentioned in papers about (F)ACT from Scandinavian countries (40, 45, 59, 60, 72). This is an enhancement of the multidisciplinarity, used in the (F)ACT program, that has been practiced and recommended for the last decades.

Intersectoral collaboration is often recommended in the literature found, but, notably, the literature found does not elaborate on what integrated collaboration should look like in practice. Possibly that is because other literature confirms that this collaboration is not easy to achieve (92). Integration can be defined as the search to connect the healthcare system (acute, primary medical, and skilled) with other human service systems (e.g., long-term care, education, and vocational and housing services) to improve outcomes (clinical, satisfaction, and efficiency). Leutz places full integration into the larger context of good human service practices by integrating services through linkage and coordination (92). Accordingly, we should not set full integration as the goal for community mental healthcare, rather, good connections and collaboration are more achievable.

In recent years, citizenship for people with (severe) mental health problems is a topic that has received increasing attention (93–95). Citizenship concerns one’s connections to the responsibilities, rights, roles, relationships, and resources offered to people in society (96). There have also been an increasing number of empirical studies, such as the cross-sectional study by Nesse and colleagues (97). This study suggests that citizenship and occupational meaningfulness may have positive implications for recovery. Additionally, Rowe and Davidson presented “recovering citizenship” as a concept and metaphor to capture the individual recovery process within the context and goal of a life in the community that the citizenship framework supports (98), and which is also about social inclusion and the full participation of individuals with mental illness in society (99).

Worthy of note is that just a few papers come from the leading journals on integrated community mental healthcare, which we explicitly searched, including the *International Journal of Integrated Care* and *Community Mental Health Journal*. Remarkably, the term “SMI” appears just once in the titles and abstracts of the volumes of 2011–2021 of the first journal. Moreover, in the *Community Mental Health Journal* we found several papers about community care, but only a few in combination with SMI. This confirms the idea that little research has been done on this topic. Besides that, many papers also seem to be written from the point of view of the mental healthcare sector. Loneliness, debts, and poverty are important topics in community care, but there does not yet seem to be much published about these main topics in social services because they did not show up in the results of the papers found. In addition, research in the shelter and supported housing sector is still limited (23, 24). We also noticed that social work as a distinct support sector alongside the mental healthcare sector that includes supported housing receives little attention in the literature found. It is recommended that there should be more attention paid in future research to mental healthcare from the community perspective in which the municipalities and social services play a larger role.

In this scoping review, we have chosen to exclude papers on interventions that focus on a specific area of life and do not provide an integrated offering for people with SMI only, because of the risk of investigating too broad a scope. In the last decade, some interventions have become an important and innovative part of community care but would be too much information to present in one scoping review. Consequently, we did not include papers about (returning to) work, and papers primarily focused on recovery. Nevertheless, interventions are the important link between theory and practice and are worth mentioning. The literature proves their importance because of the many available interventions for this target group to improve, for example, lifestyle (100, 101), internalized stigma (102), housing (103), employment (104, 105), cognition (106), social skills (107), and self-management (108). Therefore, the focus on psycho-social aspects of support for people with SMI in the included papers is limited. This may also be due to our focus on the broader literature and not on interventions that address these aspects more specifically.

Finally, it was difficult to compare the papers. One example is the difficulty of comparison in the context of national differences in legal frameworks and public policies. Not every recommendation could be implied in all societies. We tried to take this into account to some extent by including only Western literature. In addition, for both the target group and the outpatient setting, a very varied vocabulary is used in the various papers and the general terms do not mean the same in every article. First, the term “supported housing” is used for support to people in a 24/7 aggregated setting, but also with regard to clients who live independently in the community with 24/7 available support, which is what we were looking for. Second, the term “SMI” is interpreted differently. Some papers are limited to clients with schizophrenia and bipolar disorder, while other papers focus more on autism and anxiety. Other requirements for SMI are also handled differently. Several papers seem to focus more on common mental illnesses rather than SMI. This makes it hard to compare and generalize the different papers on this topic. Previous research has already indicated that varying terminology is used internationally to describe the different housing settings and approaches to the provision of housing and support (13). Further research is necessary to create general terminology with clear definitions of the outpatient setting.

### 4.2. Strengths and limitations

The main strength of our review is the broad and systematic search. We used several search strategies, including database search, hand-searching the reference lists and leading journals to find as complete an overview as possible of all papers on our topic. We have done everything possible to find all relevant papers from the past 10 years. To ensure that we did not miss innovative topics, we did not choose certain study designs as inclusion criteria. This also has the advantage that we could find enough papers. As a result, there are large differences between the study designs in the papers. This creates more difficulties when comparing the papers and ingredients. It is hard to conclude which ingredient is more important for community-based mental healthcare than another. Nevertheless, to the best of our knowledge, this is the first scoping review on all developments in community mental healthcare and gives a good overview of the current relevant topics. Notably, less than half of the included papers are empirical studies, and a large part of the included papers was composed of descriptive or opinion papers. More empirical research is needed on this subject.

Conducting a scoping review provides a broad view of the literature, but it also has some limitations. One of these includes the search terms. With these search terms, it was not possible to find everything in the field through database search due to the variation in terminology, and in recent years the main focus of the research has been on individual interventions. Despite all efforts, including the Research Rabbit software and hand-search, there is still the possibility that we missed some relevant papers. The second limitation of our study is the generalizability of the conclusions. We did not use search terms in our search strategy to find specific themes, such as citizenship and social work, but these themes are related to our search terms recovery, participation, social inclusion, and empowerment. The final limitation concerns citizenship. Despite the increasing attention being paid to citizenship in the scientific literature, we included only a few citizenship papers. Due to the target population of our broader study, we only included papers on adults with SMI while the citizenship papers focus on (common) mental illness. Nevertheless, the focus on citizenship is a relevant development that deserves attention in this scoping review.

## 5. Conclusion

This scoping review aimed to give a comprehensive overview of existing and upcoming community mental healthcare approaches to discover the current vision in the areas of ingredients. We found 12 areas of ingredients, including some innovative topics about reciprocity and sustainable policies and funding. There is much attention paid to individual ingredients for good community-based mental healthcare, but very little is known about their integration and implementation in contemporary, fragmented mental healthcare services. No earlier, international study has connected all the current elements of good community mental healthcare together. Thus, our research contributes to the existing research and adds value to future research on community-based mental healthcare. For future studies, we recommend more empirical research on community mental healthcare, as well as further investigation(s) from the social service perspective, and solid research on general terminology about SMI and outpatient support.

## Author contributions

CvG contributed to the development of the search question and strategies, screening papers and analysis, and to the main part of the manuscript. DR and MvV participated in the development of the search question and strategies, participated in screening papers, the thematic analysis, and writing the manuscript. JvW and TvR participated in the development of the search question, strategies, and supervised advancement of the project. All authors contributed to the article and approved the submitted version.

## Funding

This scoping review belongs to a broader project which received ethical approval from the Ethics Review Board Tilburg School of Social and Behavioral Sciences and was funded by three organizations for supported housing and shelter in Netherlands: Kwintes, Leviaan, and HVO Querido.

## Conflict of interest

The authors declare that the research was conducted in the absence of any commercial or financial relationships that could be construed as a potential conflict of interest.

## Publisher’s note

All claims expressed in this article are solely those of the authors and do not necessarily represent those of their affiliated organizations, or those of the publisher, the editors and the reviewers. Any product that may be evaluated in this article, or claim that may be made by its manufacturer, is not guaranteed or endorsed by the publisher.
